# COVID-19 and the irony of military expenditures: non-verbal semiotic discourse study

**DOI:** 10.1016/j.heliyon.2022.e09324

**Published:** 2022-04-25

**Authors:** Enas Naji Kadim, Ali Haif Abbas

**Affiliations:** Wasit University, Wasit, Iraq

**Keywords:** Visual irony, Semiotics, Military expenditure

## Abstract

This article presents a study in the non-verbal semiotic discourse analysis of visual ironies of military expenditures in some selected cartoons amid COVID-19 spread. Visual irony can be expressed through using humorous or sardonic cartoons or posters with written expressions to express the opposite of what is really going on. Global military expenditure reaches $1917 billion in 2019. Such massive spending on military equipment failed to fight an unseen enemy. This article aims to analyse, from a non-verbal semiotic discourse perspective, some selected cartoons related to military spending amid the rise of COVID-19 pandemic. The selected cartoons are analysed according to Peirce's triadic system of the sign. The analysis of the ironic cartoons related to military spending indicates that governments should reduce spending on military. Instead, they should focus on spending on other crucial humane, economic, and health problems.

## 1 Introduction

The World Health Organization (WHO) identified COVID-19 as a pandemic on 11 March 2020. COVID-19 is classified as a pandemic due to its danger, high number of mutations, rapid spread, infections, and deaths. COVID-19 is a respiratory illness and can also affect other humane organs ranging from mild respiratory symptoms to severe pneumonia and death (Coccia, 2020a; 2021a; 2021b). It is the third virus which belongs to the family of coronaviruses. Two other coronaviruses appeared before COVID-19. The first one was called SARS-CoV (Severe Acute Respiratory Syndrome) which appeared in 2002–2003 China. The second was called MERS (Middle East Respiratory Syndrome) which appeared in Middle East particularly in 2012, Saudi Arabia (Abbas, 2020a, Abbas, 2020b). The symptoms of the pandemic are fever, cough, and shortness in breathing. COVID-19 has influenced people's lives and caused devastating problems to our health, economic, and financial systems (Abbas, 2020a, Abbas, 2020b; Tao et al., 2021). As of February 22, 2022, there are a total of 432,981,424 COVID-19 cases with 5,937,586 deaths in the world according to the latest updates of Johns Hopkins University (https://coronavirus.jhu.edu).

COVID-19 treatment is still unavailable. The world depends on medical supplies, social distancing, white armies, vaccines, and health systems in the fight against the pandemic. In terms of health systems, the Global Health Security Index (GHS) Index is the first comprehensive assessment of global health security capabilities in 195 countries. The GHS Index measures health systems in terms of 6 categories namely, prevention (the ability to prevent the emergence of pathogens), detecting and reporting (the ability to detect and report for pandemics and epidemics of potential concerns), rapid response (rapid response to and containing of the spread), health system (sufficient and robust health systems to treat the sick people and protect health workers, compliance with international norms (commitments such as improving national capacity, financing plans to discover and bridge gaps, and adhering to biological threats), and risk environment (the total risk environment and country vulnerability to biological threats). The GHS Index analysis demonstrates that all the 195 countries are not fully prepared for epidemic and pandemic threats. International preparedness is identified as weak. Many countries do not possess good health security potentials to detect, respond, and prevent biological threats. The overall average GHS Index score is 40.2 out of 100. High income countries report an average score of 51.9 out of 100. This indicates that all international preparedness for biological threats remains weak (Global Health Security Index, 2019). Coccia (2021c) concludes that high health expenditures and low exposure of population to air pollution are critical and crucial factors that can reduce fatality rate of COVID-19 pandemic. The statistical evidence illustrates that countries with low COVID-19 fatalities have high spending on health systems. Therefore, the study recommends that one of the best and effective strategies to reduce fatalities of future pandemics is healthcare enhancement and environment sustainability in society. Coccia's (2022) study in the preparedness of countries to face COVID-19 demonstrates that all countries have weaknesses and have not high preparedness to face or fight future epidemics or pandemics. The results of the study illustrate that the best countries to face COVID-19 crisis have smaller size of population and better public government associated with high health expenditures. COVID-19 and other future pandemics are problems which cannot be solved by research in medicine only. Other parts of the solution of current and future biological threats are advanced capabilities and technologies for reducing air pollution and increase of renewable energy Coccia, 2020b).

World military expenditure is massively increasing once again. Estimated global military spending reaches $1917 billion in 2019 which is the highest level since 1988 (SIPRI; Nan et al., 2019). This massive number of military spending is 3.6 percent higher than that of 2018 military spending and 7.2 percent higher than that of 2010 military spending. The top five military spenders are: the United States, China, India, Russia, and Saudi Arabia which together account for 62 percent of global military spending. The subtotal top five military spenders are: France, Germany, United Kingdom, Japan, and South Korea. The subtotal top ten spenders are: Brazil, Italy, Australia, Canada, and Israel. The subtotal top fifteen spenders are: Turkey, Spain, Iran, Netherlands, Poland, Singapore, Taiwan, Algeria, Pakistan, Colombia, Kuwait, Indonesia, Iraq, Thailand, Norway, Oman, Mexico, Sweden, Greece, Ukraine, Chile, Switzerland, Romania, Belgium, and Denmark (Nan et al., 2019).

Undoubtedly, military expenditure is necessary for international economy. Definitely, countries need to secure their borders and be ready to deal with any internal or external threats. But any resource use carries an opportunity cost. In other words, military equipment costs money. These amounts of money can be employed and used on other resources and projects that can improve the pace of development and economy (Dunne and Uye, 2013). The empirical analyses, of whether military spending has effects on economy, demonstrate that there are little or no positive impacts on economic growth, on the contrary, military spending has negative impacts, or at best no significant impacts on economic growth (Mylonidis, 2008; Dunne and Uye, 2010). Military expenditures impede the economic growth and cause reductions in investments and exports (Heo, 1999). Military spending reduces the amount of resources that can be used to develop economy.

In their article entitled “Military Expenditure and Economic Growth: A Survey”, Dunne and Uye (2013):

add 65 studies—bringing the total to 168 surveyed studies—and find that the more recent studies provide increasingly strong evidence of a negative effect of military expenditure on economic growth. It also finds that cross-country studies that use a relatively large amount of post-cold war data are more likely to find negative effects (8).

Previous studies demonstrated that military spending negatively influences economy and thus growth. For more information see (Smith, 1977, 1978, 1980; Lim, 1983; Landau, 1985; Mintz and Huang, 1990; Ram, 1995; Dunne et al., 2002).

Undoubtedly, military expenditures can advance and raise as well as downgrade and reduce growth. Military expenditures might promote growth through developing new technology which can be used in private sectors. Military expenditures may create socioeconomic structure through spin-offs effects; provide security and protections against threats, and increase aggregate demand and employment through the Keynesian multiplier effect. On the contrary, military spending has negative impacts on growth through its costs, gun-butter tradeoff on one hand, and its negative impacts on investment and other productive activities on the other. Governments often spend high amount of money on military equipment and this high spending comes with high government debt and increased tax burden. This definitely reduces growth (Awaworyi and Yew, 2018).

The aforementioned countries and many others have stood helpless unable to control the outbreak with all their sophisticated military equipment. This rise of military expenditure raises the following questions: what is the purpose behind this massive increasing? Does it mean that the world is witnessing a catastrophic security problem? Can spending large amount of money on military equipment solve such a security problem? Can such expensive military equipment kill an unseen enemy that has killed millions of people since its outbreak on 31 December, 2019? Some selected cartoons ironically answer all of these questions. Based on the analysis of the selected cartoons on military expenditures and COVID-19, the article showed that the world's insane spending on military is unable to fight the pandemic. All different types of sophisticated and nuclear weapons cannot fight an unseen virus. A simple face mask costing a dollar is fighting the pandemic better than a nuclear bomb costing billions of dollars. Therefore, the study recommends that the world should reduce spending on military and shift its focus to other crucial health, environmental, educational, and social problems.

## 2 Semiotics and cartoons

Communicating messages and information can either be through words whether spoken or written or through other non-verbal means such as body language, signs, and gestures. The former is called verbal discourse, while the latter is called non-verbal discourse. Cartoons have widely been used in politics, media, and election campaigns. A cartoon is a crucial way of conveying or communicating important ideas, sensitive issues, deep meanings and thoughts to lay people. Cartoons can be classified as either simple or complex. Some cartoons do not need good background knowledge and analytical skills to understand and comprehend their hidden messages, while some others need good background knowledge and analytical tools in order to understand their hidden messages (Nelson, 1975; Lent, 2000; Diamond, 2002). Irony, satire, humor, hyperbole, labeling, and analogy are used in cartoons to convey certain messages about different issues or problems in a society. Most of cartoons are ironically and satirically used to criticize people, especially those in power and their actions (Shaikh et al., 2016).

Signs cannot be separated from our current world. Signs can be seen everywhere; in buildings, streets, companies … etc. they stand for someone or something and convey different messages and meanings (Sebeok, 1994). Everything can be called a sign. In other words, a picture is a sign, crying, pointing finger, winking, laughing are all signs. Therefore, cartoons are also signs carrying different messages and meanings that are helpful in our daily life (Shaikh et al., 2016). Over the past few decades, the power of the sign (visual power) has been given more attention in the humanities, social, and political sciences (Pham, 2013). Semiotics is derived from the Ancient Greek word “sēmeion” which broadly means “sign”. Semiotics is the science that deals with all the signs and symbols that can be used to communicate a message. It is served as a crucial theoretical and analytical framework for the analysis and interpretation of all the signs and symbols that are used in communication such as language, words, sounds, images, music, advertising, painting, and fashion (Kuzu, 2016). A cartoon is a drawing or painting intended to influence people or societies about some people, ideas, problems, and situations. According to Sanaky (2013:100), cartoons contain visual images, symbols, and sometimes written texts often used in the form of satire to convey a message of attitude towards certain societies, groups, people, events. Most cartoons are used for criticizing intolerance, injustice, political corruption and social evils (Putri, 2018).

A cartoon is a set of signs which are used to communicate a message and elicit laughter (AlShurafa et al., 2021). A cartoon can be described as a genre of its own. It is composed of a well-organized multimodal structure which consists of visual components based on a broad spectrum of representational styles. It is sometimes flavoured with verbal texts. It attempts to convey a set of beliefs or ideas through using a critical message in a mild humour or sarcastic tone (Chu, 2020). Zhang (2021) studies the political cartoons of the US-Sino trade war which is based on three perspectives namely, semiotics, cognitive, and cultural. All political cartoons are used for persuasiveness by using emotional source domains/vehicle concepts, but for different audiences. Alkhresheh (2020) conducted a study on the editorial cartoons of the Pakistani newspaper namely, Dawn and the British newspaper namely, The Economist on COVID-19. The study showed that the Dawn focused more on the severe attacks of COVID-19 and the effects of locusts on the agriculture-based economy, while The Economist focused on the American cultural disorder of racism, the difficult situation of Brazil due to its insufficient solutions of the government to control the spread of COVID-19.

## 3 Method

This section is concerned with the selection and description of the selected cartoons on one hand and the presentation of the selected analytical framework for the analysis of the selected figures on the other.

The search engine expressions are namely, COVID-19 and military expenditures cartoons, military spending and COVID-19 cartoons, military budget and COVID-19 cartoons. After searching the Google, we only found four cartoons related to the irony of military expenditures and COVID-19. Therefore, we analysed the cartoons according to Peirce's triadic system of the sign which is clearly presented below. The cartoons are concerned with the irony of military spending and its failure to fight the pandemic which has taken millions of innocent lives. The figures are not analysed in a chronological order. They also do not target a specific country. They are general visual ironies criticizing the high spending on military and the low spending on the healthcare systems of all countries. Due to copyrights and our inability to get permission to reuse the images (cartoons) from the copyright holder, we decide to remove the images from the analysis. But all the details of the images including author, title, and link of each image are mentioned in the references section. The first image is taken from Desai (2020). The second image is taken from Broek (2020). The third image is taken from Luckovich (2020), and the fourth image is taken from Hallinan (2020).

The pandemic teaches us a lesson and we should not ignore this lesion; instead we should learn from this lesson by developing and enhancing our health systems for next future epidemics or pandemics. The websites of the analysed cartoons are presented in the reference sections.

During the nineteenth century and later, two important schools played a vital role in the field of semiotics: the European school and the American school (Abbas and Kadim, 2019). The European school is represented by the Swiss linguist Ferdinand de Saussure (1857–1913) and his followers. The American school is represented by the American philosopher, Charles Sanders Peirce (1839–1914) and his followers. Saussure and Peirce are founders of modern semiotic theories. Their theories have set the basic principles for describing and interpreting any sign. Most post-Saussurian and post-Peircean studies on semiotics have adopted their theories of the sign (Yakin and Totu, 2014; Abbas and Kadim, 2019).

**Figure 1:**
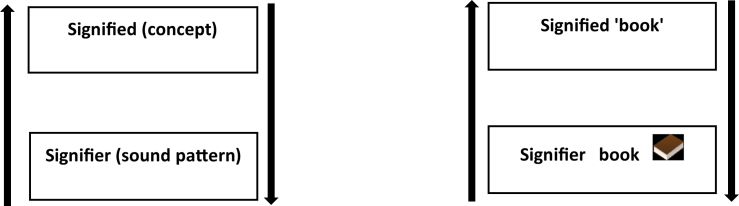
Saussure's dyadic system of the sign (Adopted from Abbas, 2020a, Abbas, 2020b: 2276).

Saussure's dyadic theory of the sign is formed by the relation between a signifier (sound pattern) and a signified (concept). The signifier is the perceptible form of the sign (for example, the visual appearance of a street sign). The signified is a certain mental idea for which the perceptible form stands. For example, the visual appearance of the wordbook (signifier) stands for the concept of a book (the signified). The signifier and the signified are interrelated and they both form the sign. The relation between the signifier and the signified is arbitrary. In other words, there is no logical connection between the concept of a book and its visual appearance (Abbas and Kadim, 2019: 189) (see Figure 1).

**Figure 2:**
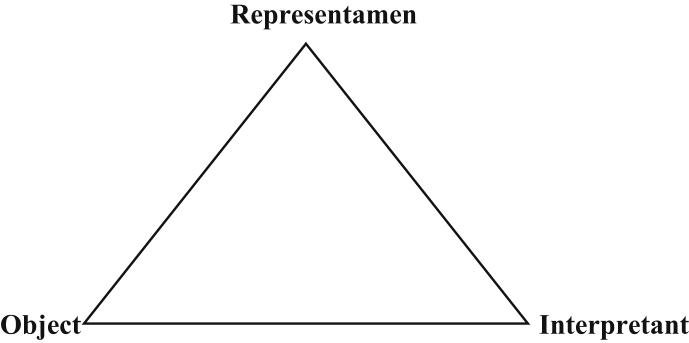
Peirce's triadic system of the sign theory (Beebe, 2004: 637).

Peirce's triadic system of the sign consists of three parts: the representamen (the physical shape of the sign) is similar to the signifier in Saussure's dyadic sign theory, the object (the sign vehicle- it determines the sign). The object is the referent to which the sign refers to, and the interpretant (the meaning or understanding of the relation between the sign and the object it signifies) (Siau and Tian, 2009) (see Figure 2).

Tear, for example, is a sign. It is the representamen in Peirce's triadic system. In terms of object, tear is water comes out from the eyes when someone is in a state of high emotion whether feeling happy or sad. In terms of interpretant, the tear is associated with happiness or sadness (see Table 1).

**Table 1:** Semiotic process (Adopted from Aryuni, 2012: 29).

Representamen	Object	Interpretant
Tears	Water which coming out from eyes when someone is in a state of high emotion, whether sad or happy. Or when the eyes of someone possessed by dust or injured.	Very sad, weepy, hurt, scared, sensitive or very happy

The figures are analysed according to Peirce's triadic system of the sign namely, representamen, object, and interpretant.

## 4 Results and discussion

This section is concerned with the analysis of the selected cartoons about the irony of military expenditures and COVID-19. The cartoons are analysed according to the three semiotic processes namely, representamen, object, and interpretant. The first image (cartoon) is taken from Desai (2020).

The cartoon consists of three figures: the man looks like a soldier heavy loaded with different weapons and military equipment. An angry face monster with a head full of spikes similar to a crown is holding our planet earth with his huge hands. A small doctor wearing a face mask and catching a stethoscope is standing between them. In addition to the three figures, three written expressions are clearly presented in the cartoon. The expression “defense budget” refers to the heavy loaded soldier. The second expression ‘healthcare budget’ refers to the doctor with a simple surgical face mask and a stethoscope. The soldier is pointing with his right hand to the doctor and telling him to “Go. Save the world!”

The cartoon is full of dramatic ironies, ironies that we all know, but we unfortunately neglect their value: the first irony belongs to the heavy loaded soldier who with all of his expensive and sophisticated weapons and military equipment cannot kill the monster or coronavirus. The verbal expression “Go. Save World!” which is uttered by the heavy armed soldier is ironic. Although the soldier is heavy loaded with weapons, he could not kill the monster; instead he is ordering a small doctor with a simple surgical mask and a stethoscope to free our planet from the unmerciful hands of the monster (coronavirus). The little simple and weak medical supplies in comparison with the expensive and sophisticated military equipment are ironic. The surgical mask and the stethoscope are not the suitable weapons one can use to fight a deadly virus. They are similar to one fighting a fighter aircraft with a stone. The various expensive weapons which the soldier is holding are also ironic and catastrophic. The weapons which cost billions of dollars could not kill the virus and defend the world. The weapons also refer to the countries which are spending hundred billions of dollars on defense budget neglecting the scientific and healthcare and other crucial budgets for our security. COVID-19 is personified as a dangerous monster occupying our entire planet. The soldier refers to global military spenders, especially the high income countries which fail to fight an unseen enemy (see Table 2).

**Table 2:** Semiotic processes in the first visual irony.

Representamen	Object	Interpretant
The soldier	One engaged in the military service	Frightened, horrified, terrified, scared, coward unable to fight
The doctor	Medicine man	Associated with healing illness and keeping away evil spirits
Monster	An angry face monster with a head full of spikes	Corona virus, disease, danger, infections, deaths
weapons	Any object used in fighting or war	A symbol of failure, failure to fight the enemy
mask, Stethoscope	Mask: a protective covering for the face or part of the face. Stethoscope: a piece of medical equipment that doctors use to listen to your heart and lungs.	Although they can fight corona better than the sophisticated weapons of the soldier, but they are associated with weakness, inadequateness, and insufficiency in our healthcare systems
Planet Earth	The planet on which we live that is third in order from the sun	Associated with danger, destruction, annihilation, devastation, ruin, and wreckage

The second cartoon is taken from Broek (2020). The cartoon consists of many different and sophisticated weapons on its right edge. The weapons are varied from land forces which are represented by the tanks, air forces which are represented by the aircraft and sea forces which are represented by the huge battleship. Different huge missiles can be clearly seen in the cartoon. A written expression entitled “Disease's war” is listed clearly on the top left of the cartoon. In the middle-left part of the cartoon, many angry faces with spikes similar to a crown prince can be clearly seen. On its bottom left, two animals are looking at the weapons. The first one is asking: *do they fight Covid-19?* The other one is answering: *they will fail, I presume*.

The first think that should be noted in the cartoon is the written expression “Disease's war”. The world is at war against a disease. This war is not similar to the First World War or the Second World War or a civil war. It is a new type of war having its own weapons, ways, and plans of dominating the planet. It has no allies. All people are enemies. It shows no respect for human rights. It violates the rules of international humanitarian law. It shows no mercy for children, women, and old people. It is a dangerous enemy that cannot be killed by a gun or a tank or an aircraft, or a battleship. Even the intercontinental ballistic missiles cannot kill or defend the world against the fierce attacks of the disease. The dramatic irony which all people know is that all the weapons which cost the world billions of dollars cannot kill this disease. The panicked commander who is standing on the battleship is associated with fear, bewilderment, helplessness, powerlessness, and weakness. His scary face reflects a helpless world unable to fight a virus. The various sophisticated weapons which clearly appear in the cartoon are an irony of failure, failure to fight an unseen enemy that is taking hundreds lives every day. The angry faces of corona virus with their poisonous spikes like the crown are associated with war, fierce attack, annihilation, and extermination, extermination of our beloved ones. The choice of the two animals in the bottom left of the cartoon is ironic. There is a tendency to show that even animals know the fact that weapons and military equipment which cost billions of dollars will definitely lose the fight against COVID-19 (see Table 3).

**Table 3:** Semiotic processes of the second visual irony.

Representamen	Object	Interpretant
Weapons	Any object used in fighting or war	Associated with failure, failure to fight an unseen enemy that is taking hundreds lives everyday
Angry faces	Coronavirus (COVID-19)	Associated with war, fierce attack, annihilation, and extermination, extermination of our beloved ones
Commander	An officer who is in charge of a military operation	Panic, fear, bewilderment, helplessness, powerlessness, and weakness
Two Animals	An animal is something that lives and moves but is not a human	Some animals know the fact better than the people, especially those in charge

The third cartoon is taken from Luckovich (2020). Different huge and sophisticated weapons can be clearly seen on the left part of the cartoon. A commander is standing on the battleship and looking down to the woman. The woman is raising her right hand, pointing with her finger and politely requesting “May I use that flag to make a face mask? A listed written expression on the battleship entitled “$ 700 billion-defense budget”.

In terms of the argumentative analysis, the various sophisticated weapons are a visual irony of massive spending on military. The written expression “$ 700 billion-defense budget” is a written irony used to refer to the massive spending on military. The woman who is pointing her finger to the flag and asking permission to make from the flag a face mask is dramatic. The woman left all the huge and expensive weapons and pointed to the flag to make from it a tool of protection. All the weapons and military equipment in the cartoon are useless. They cannot help the woman or protect her. The face mask is a protective covering which goes over part of one's face. The face mask is made from clothes. There is a tendency to show that bullets or tanks or missiles cannot fight such type of enemy. Among all these sophisticated and expensive weapons, a piece of clothes costs some dollars can only help (see Table 4).

**Table 4:** Semiotic processes of the third visual irony.

Representamen	Object	Interpretant
Weapons	Any object used in fighting or war	Associated with failure, failure to fight an unseen enemy that is taking hundreds lives everyday
A woman	An adult female person	Doubt, incertitude, no confidence, uncertainty
commander	An officer who is in charge of a military operation	bewilderment, helplessness, powerlessness, and weakness
A flag	A piece of cloth with a special design and colours that is the symbol of a country	A symbol of protection against the disease which can be effective more than weapons and military equipment

The fourth cartoon is taken from Hallinan (2020). Angry faces with spikes similar to a crown are attacking and destroying a big military vehicle. A clear and capitalized white written expression entitled “Global Military Spending” can be seen listed on the military vehicle. On the right part, a big vehicle can be seen with a red cross and a large white written expression. The written expression entitled (Spending on Healthcare and Social Safety-Nets”. Other written expressions can be clearly seen in the cartoon. The written expression “Post-Covid World” is listed on the top. On the right top of the cartoon, three written expressions are listed: Bioweapons, Biological Threats, and Pandemics.

This cartoon conveys many crucial messages to the world. One important message is that pandemics do not know borders or distinguish between black people and white or Christians and Muslims, male or female, men or women, old or young. All people are victims to pandemics. This is a fact that we all should know especially politicians and leaders. The insane global military spending cannot win the battle against dangerous pandemics like COVID-19. As it can be seen in the cartoon, the angry faces with the poisonous coronary spikes are destroying the military vehicle. There is a tendency in the cartoon to say that an unseen virus is destroying the whole world military spending. The world military spending is standing helpless in front of an unseen enemy. The red colour of coronavirus is associated with energy, victory, angry, blood, and murder, murder of the innocent people by corona. The cartoon from the right is rather optimistic serving as a moral lesson that we should learn and never forget. The world should spend money on healthcare systems and social safety. It gives us a future plan after COVID-19 world, a world full of healthcare equipment and supplies rather than military equipment, a world that should pay more attention to pandemics, biological weapons, biological threats rather than civil wars and regime change. Denotatively, the red cross refers to the international organization that helps people who are suffering because of a war or natural disaster. Connotatively, the red cross is associated with protection, safety, impartial assistance to all people, regardless their race, religion or citizenship status (see Table 5).

**Table 5:** Semiotic processes of the fourth visual irony.

Representamen	Object	Interpretant
Angry faces with spikes	Coronavirus (SARS-CoV-2)	Associated with rage, murder, destruction of our beloved people and planet.
Military vehicle	An object used in fighting or war	Associated with failure, failure to fight an unseen enemy that is taking hundreds lives every day.
Healthcare vehicle	An object used to treat and save the lives	Associated with success, success to fight epidemics and pandemics.
Post-Covid World	A written expression	A future plan and a moral lesson that we should reduce spending on military and pay more attention to our healthcare systems.

## 5 Conclusions

This article presented a study in the nonverbal-semiotic discourse analysis of selected cartoons on military expenditures and COVID-19. Massive military expenditure is paradoxical since it takes place in a world with less military threats. In other words, we should shift our focus on the threats and challenges that need no military equipment and highly sophisticated weapons (Sköns, 2005) such as the one that we are facing right now, namely COVID-19. Governments should reduce high spending on military and focus more on human, social, educational, scientific, and medical developments (Apanisile and Okunlola, 2014). The analysed cartoons succeeded in conveying ironically the general fact of the massive military expenditures that our planet is witnessing today. Instead of this massive military spending, the world should more focus on other catastrophic problems that are threatening our existence such as climate change, global warming, and pandemic mutations and developments. The study is limited to the semiotic analysis of four figures on the irony of military expenditures. Therefore, the study encourages that more research should be done on the effects of high military spending on social, health, environment problems. The study is an attempt to motivate scientists, experts, academicians, World Health Organization, and other health and scientific organizations to conduct more research on the necessary need for high spending on health systems and recommendations for fighting future diseases. We list the following recommendations below:

1. Governments need to reduce spending on military.
2. Governments need to increase spending on health.
3. According to the GHS Index, the world is weak in fighting current diseases and future diseases and pandemics. Therefore, governments and decision makers must learn from the current lesson of COVID-19 paying more attention to future threats in terms of prevention, detection, reporting, rapid response, health systems, compliance with international norms, and risk environment.
4. All the world's health systems are weak in response to pandemics. For this reason, we need to enhance our health systems by looking for the current weak points and bridge the gaps with immediate solutions based on scientific research and studies.
5. Health and environment are also interrelated. Therefore, we need to secure our planet form pollution, global warming, and climate change threats.
6. Achieving security and stability along with the health systems are also crucial and necessary. We should avoid wars and struggles. The Yemeni conflict is one current example. After more than five years of increasing conflict, Yemen's health system is on the brink of collapse (WHO. https://www.who.int/emergencies/situations/yemen-crisis). After the ten years of the Syrian crisis, Syria health system has been devastated, up to 50% of the health infrastructures have been destroyed, and up to 70% of health workers have fled the country looking for safety and peace (Alhaffar and Janos, 2021). Currently, we are very sad in what is happening between Russia and Ukraine.
7. Technology and artificial intelligence along with medical workers are crucial and effective in the fight against diseases. Therefore, governments should invest more money on developing technologies to join the fight against future diseases, not to develop technologies to make weapons of mass destruction.

## Declarations

### Author contribution statement

Enas Naji Kadim and Ali Haif Abbas: Analyzed and interpreted the data; Wrote the paper.

### Funding statement

This research did not receive any specific grant from funding agencies in the public, commercial, or not-for-profit sectors.

### Data availability statement

Data included in article/supplementary material/referenced in article.

### Declaration of interests statement

The authors declare no conflict of interest.

### Additional information

No additional information is available for this paper.
